# Drevogenin D prevents selenite-induced oxidative stress and calpain activation in cultured rat lens

**Published:** 2007-07-12

**Authors:** P.G. Biju, B. N. Rooban, Y. Lija, V. Gayathri Devi, V. Sahasranamam, Annie Abraham

**Affiliations:** 1 Department of Biochemistry, University of Kerala, Kerala, India; 2 Regional Institute of Ophthalmology, Medical College, Kerala, India

## Abstract

**Purpose:**

Selenite-induced cataractogenesis is mediated by oxidative stress, accumulation of calcium and activation of lenticular calpains. Calpains are a super family of Ca^2+^ dependent proteases, which are involved in lens protein proteolysis and insolubilization. Many inhibitors could prevent calpain-induced proteolysis of α- and β-crystallins in rodent cataracts. Evaluating natural sources with antioxidant property and subsequent prevention of calpain activation may lead to the development of safer and more effective agents against cataractogenesis. There are no reports on the protective role of bioactive components against calpain-mediated proteolysis and subsequent cataractogenesis. The purpose of the study was to evaluate the role of Drevogenin D, a triterpenoid aglycone, isolated from *Dregea volubilis* in preventing selenite-induced, calcium-activated, calpain-mediated proteolysis in cultured rat lenses.

**Methods:**

Lenses were extracted from Sprague-Dawley strain rats at the age of one month and were organ cultured in M-199 medium with HEPES buffer. The lenses were divided into three groups with eight lenses in each group as follows: lenses cultured in a normal medium (GI), lenses cultured in a sodium selenite supplemented medium (GII), and lenses cultured in a medium supplemented with sodium selenite and Drevogenin D-treated (GIII). Changes to transparency and opacity formation of lenses were monitored under microscopic observation. At the end of the experiment, biochemical parameters such as activity of lens superoxide dismutase (SOD), lens Ca^2+^ ATPase, concentration of Ca^2+^, levels of sulfhydryl content, and thiobarbituric acid reacting substances (TBARS) were determined. Changes to casein zymography for calpains, immunoblot for Lp82, and SDS-PAGE of lens water soluble protein fraction (WSF) were also done.

**Results:**

Microscopic evaluation of lens morphology showed that Drevogenin D prevented the opacification in G-III. Drevogenin D inhibited the accumulation of calcium, the activation of calpain system, and lipid peroxidation. Activity of Ca^2+^ATPase, SOD, and SDS-PAGE profile of water soluble proteins was normalized following treatment with Drevogenin D.

**Conclusions:**

Selenite-induced cataractogenesis is mediated by oxidative stress leading to a decrease in the activity of Ca^2+^ ATPase, resulting in the accumulation of calcium and the subsequent activation of lenticular calpains. The results obtained indicated that Drevogenin D treatment was effective in protecting the lens proteins by controlling stress-induced protein oxidation, maintenance of Ca^2+^ ATPase activity, calcium accumulation, lipid peroxidation, and prevention of calpain activation. Hence, Drevogenin D can be used as a potential therapeutic agent against oxidative stress-induced cataract.

## Introduction

Cataract remains the major cause of curable blindness, accounting for more than half of the total blind [1] and its prevalence in developing countries is much more than in developed ones [2]. Causative factors of cataract are many and its etiology is unclear, but oxidative stress is thought to be one of the underlying factors of most cataracts [3]. As of now, modern medical science has no effective treatment for cataract except surgery, though effective surgical remedy has its own limitations. Development of a drug which could prevent or delay the onset of cataract will lessen this burden and reduce the number of sightlessness.

Various experimental models have been used to study the etiology of cataract and to evaluate therapeutic effects. Selenite-induced cataract has been proven to be an extremely rapid and convenient model for nuclear cataracts. Cataract is induced in suckling rat pups by an overdose of the essential trace element selenium [4]. Several biochemical processes such as oxidative stress, altered epithelial metabolism, calcium accumulation, calpain-induced proteolysis, crystallin precipitation, phase transition, and cytoskeletal loss occur during the development of selenite-induced cataract. Of the above, ionic homeostasis is a key factor for maintenance of lens transparency. Loss of Ca^2+^ homeostasis has been implicated in most types of cataract [5,6]. Levels of Ca^2+^ are maintained in the sub-micromolar range in the cytoplasm by membrane Ca^2+^ pumps [7], plasma membrane Na^+^/Ca^2+^ exchangers [8], and endoplasmic reticulum Ca^2+^ pumps [9]. Selenite cataractogenesis is found to have increased Ca^2+^ uptake and is highest in the nucleus [10]. An important consequence of calcium elevation in lens is the activation of calpains [9]. Calpains (EC 3.4.22.17) are a family of non-lysosomal cysteine proteases with a neutral pH optimum and a requirement of calcium for activation. Calpains are widely distributed in animal tissues where they are involved in a variety of cellular processes involving calcium. Calpains consist of the ubiquitous calpains 1 (μ-calpain), 2 (m-calpain), 3 (muscle specific calpain/p94) etc. In addition to this, there are tissue-specific calpains such as lens-specific Lp82 and Lp85 in rodent lens, which are shorter splice variants of calpain 3 [11,12]. Studies on experimental cataract have demonstrated calpain-induced proteolysis of β-crystallin as a major mechanism in the lens maturation as well as cataractogenesis [13]. Lp82 is the dominant isoform of calpain in rodent lens, suggesting that it may be responsible for the proteolysis attributed to calpains in experimental cataract. Alterations to membrane proteins, lipid integrity, and the consequent increase of membrane ion permeability of the lens fiber cells have been reported in different pathological conditions [14,15]. Thus, selenite-induced oxidative stress and the subsequent loss of Ca^2+^ homeostasis are responsible for the activation of lens calpains, which results in proteolytic precipitation and aggregation of soluble proteins to insoluble proteins. Naturally occurring compounds like flavonoids, tannins, anthocyanins, terpenoids etc. are known to exhibit antioxidant activity and hence might be of potential therapeutic value [16,17]. Previous studies by our research group have clearly demonstrated flavonoids isolated from *Emilia sonchifolia* to exhibit good antioxidant and anticataractogenic potential [18].

*Dregea volubilis* Benth. ex. Hook. f. belongs to the family Asclepiedaceae and is a tall woody climber growing wild in hotter parts of India. The whole plant extract has been traditionally used to treat several diseases including eye ailments [19]. *D. volubilis* has been subjected to phytochemical analysis and found to contain several triterpenoid glycosides. Many aglycones including Drevogenin D, an aglycone of volubiloside A have been isolated from *D. volubilis* [20]. Our previous studies have shown that Drevogenin D exhibit antioxidant and anticataractogenic activity in vivo (data under publication). In the present study, we have investigated the role of Drevogenin D in preventing oxidative stress, activation of calpains, and the subsequent proteolysis of lens soluble proteins in an in vitro selenite cataract model.

## Methods

### Materials

M-199 (TC 199) culture medium, fetal bovine serum (FBS), antibiotic-antimycotic solution, sodium selenite, anti-Lp82 antibody, all chemicals for SDS-PAGE, and buffer salts for immunoblot were purchased from Sigma Chemical Company, St. Louis, MO. Alkaline phosphatase-conjugated secondary antibody, BCIP/NBT (5-bromo-4-chloro-3- indolyl phosphate/Nitro blue tetrazolium) substrate, and Tween 20 were bought from Genei, Bangalore, India. Nitrocellulose membrane (0.45 μm), Mini-Transblot, and Mini-Protean electrophoresis apparatus were from BioRad, Hercules, CA. Culture plates were acquired from Axygen Scientific, Union City, CA. All other chemicals and solvents were from SRL, Mumbai, India.

### Specimen collection

Fresh leaves of *D. volubilis* were collected locally during early summer, and the specimens were stored for reference. The specimen was verified with the herbarium of Tropical Botanical Garden & Research Institute (TBGRI), Thiruvananthapuram, India (Accession No.16591 P.S Jyothish) and authenticated by an expert (Dr. Valsaladevi, Curator, Department of Botany, University of Kerala). Specimen herbarium was submitted to the Department of Botany, University of Kerala and specimen voucher number obtained (KUBH-5582-Biju. P.G).

### Isolation of Drevogenin D

Drevogenin D was isolated from *D. volubilis* using the method of Yashimura et al. [20] with modifications. Briefly, shade dried leaves were crushed, extracted with petroleum ether (60-80 °C) to remove the sticky and fatty materials and then extracted with hot methanol. The concentrated methanol extract was hydrolyzed using 1 M HCl for two to six h and neutralized then the solid matter was dried and extracted with ether. The extract was also dried and subjected to column chromatography using silica gel G. Successive elutions were carried out with hexane, followed by various ratios of hexane-chloroform mixtures beginning with nine parts hexane to one part chloroform (9:1) with stepwise decrease in hexane and increase in chloroform ratio (8:2, 7:3, 6:4, 5:5, 4:6, 3:7, 2:8, 1:9), chloroform alone, and chloroform-methanol mixture (10:1), respectively. The fraction obtained in hexane:chloroform (1:9), was crystallized into a colorless crystalline compound with a melting point of 227-230 °C. The elemental analysis revealed the molecular formula C_21_H_34_O_5_ and answered the Libermann-Burchard reactions for terpenoids. The compound was identified as Drevogenin D (3β, 11β, 12β, 14β, 20ζ- Pentahydroxy δ^5^ pregnene) from its physical and spectral data and by direct comparison with an authentic sample using mixed melting point (m.m.p), combined infra red spectrum (co-IR), and combined thin layer chromatography (co-TLC) analysis.

### Lens extraction

Lenses were extracted through a posterior approach from the eyes of one-month-old female Sprague-Dawley strain rats under deep anesthesia. Rats used for the study were obtained from the animal house stock of the host department and handled in accordance with the guidelines as per the host's "Institutional Animal Ethical Committee" and CPCSEA (Committee for the Purpose of Control and Supervision of Experiments on Animals) rules.

### Culture Conditions

Lenses were organ cultured in M-199 medium with HEPES buffer, supplemented with 10% fetal calf serum (FCS), 100 U/ml penicillin, 0.1 mg/ml streptomycin, and 0.25 μg/ml amphotericin under 5% CO_2_ at 37 °C in a CO_2_ incubator. Selenite medium was prepared by adding sodium selenite to the medium to give a final concentration of 0.1 mM. Lenses were maintained in a 24 well culture plate with 2 ml medium/well and one lens/well for five days. Lenses developing opacification in the first 24 h were discarded.

### Grouping

Assay concentration of Drevogenin D was prepared by dissolving 5 mg/ml dimethyl sulphoxide (DMSO) and given at a concentration of 50 μg/ml medium. Lenses were maintained in normal and selenite-supplemented medium and were grouped as follows with eight lenses in each group: G-I; lenses cultured in a normal medium, G-II; lenses cultured in a selenite-supplemented medium (0.1 mM), and G-III; lenses cultured in a medium supplemented with sodium selenite (0.1 mM) + Drevogenin D-treated (50 μg/ml). Drevogenin D treatment to G-III was from the second to the fifth day while selenite administration to G-II and G-III were done on the third day.

### Assay of superoxide dismutase activity

The activity of superoxide dismutase (SOD) in the lens samples was measured by the method of Kakkar et al [21]. The assay mixture contained 1.2 ml sodium pyrophosphate buffer (0.052 M, pH 8.3), 0.1 ml of 186 μM phenazin methosulfate (PMS), 0.3 ml of 300 μM 4-nitro-blue tetrazolium chloride (NBT), 0.2 ml of 780 μM NADH, 1.0 ml homogenate (lens homogenized in 0.25 M sucrose buffer), and water to a final volume of 3.0 ml. The reaction was started by the addition of NADH and incubated at 30 °C for one min. The reaction was stopped by the addition of 1.0 ml glacial acetic acid and the mixture stirred vigorously. 4.0 ml n-butanol was added to the mixture and shaken well. The mixture was allowed to stand for 10 min and centrifuged then the butanol layer was taken out and the absorbance was measured at 560 nm against a butanol blank. A system devoid of enzymes served as the control. One unit activity is defined as the enzyme concentration required for inhibition of chromogen production by 50% in one min.

### Assay of Ca^2+^ ATPase activity

The activity of Ca^2+^ ATPase in the lens samples was measured by the method of Rorive and Kleinzeller [22]. To the reaction tube, 0.25 ml of substrate (40 mM ATP in 0.4 M Tris-HCl buffer, pH 7.4) and 0.1 ml of lens homogenate was added. A tube devoid of the homogenate served as a control. All the tubes were incubated for 30 min in a water bath at 37 °C. The incubation was stopped by adding 2 ml of 10% trichloroacetic acid (TCA) then 0.2 ml ATP were added to control tubes and these tubes were subsequently kept in ice for 20 min. All the tubes were then centrifuged at 2500x g for 10 min and the supernatant was collected. The protein-free supernatant was analyzed for inorganic phosphate. For this, 3 ml of the supernatant were treated with 1 ml of ammonium molybdate and 0.4 ml of 2, 4 aminonapthol sulphonic acid (ANSA). The color developed was read at 680 nm after 20 min.

### Estimation of levels of Ca^2+^

The levels of calcium ions in the lenses were estimated as follows. Individual lenses were weighed and digested in concentrated nitric acid:perchloric acid (5:1). After complete digestion, the samples were dried, diluted with 1% nitric acid, and made up to 50 ml in a standard flask. The samples were analyzed by flame photometry and the results were expressed as μM/g wet tissue.

### Estimation of protein sulfhydryl content

The sulfhydryl content of lens proteins was determined using the Ellman's procedure as modified by Altomare et al [23]. Aliquots of total lens homogenate of approximately 3 mg of protein were treated with an equal volume of 4% sulfosalicylic acid (SSA). The pellets obtained after centrifugation were washed with 1 ml of 2% SSA to remove free thiols. The washed pellets were dissolved in 0.2 ml of 6 M guanidine (pH 6.0) and read spectrophotometrically at 412 nm and 530 nm before and after 30 min incubation in the dark with 50 μl of 10 mM 5.5'-dithiobis-(2-nitrobenzoic acid; DTNB). Content of protein sulfhydryls was calculated using a calibration curve prepared with reduced glutathione.

### Estimation of the level of thiobarbituric acid reactive substance

The concentration of thiobarbituric acid reactive substance (TBARS) in the lens samples was estimated by the method of Niehaus and Samuelsson [24]. Briefly, lenses were homogenized in 0.1 M Tris-HCl buffer (pH. 7.5). One ml of the homogenate was combined with 2 ml of TCA-TBA-HCl reagent (15% trichloroacetic acid [TCA] and 0.375% thiobarbituric acid [TBA] in 0.25 N HCl) and boiled for 15 min. A precipitate was removed after cooling by centrifugation at 1000x g for 10 min and absorbance of the supernatant was read at 535 nm against a blank without tissue homogenate.

### Casein zymography

Calpain activity was studied zymographically by the method of Raser et al. [25]. Eight-percent gels (1 mm thickness), co-polymerized with 0.1% alkali-denatured casein, were pre-run with the zymography running buffer containing 25 mM Tris (pH 8.3), 192 mM glycine, 1 mM EGTA, and 1 mM dithiothreitol (DTT) for 15 min at 4 °C. Each sample was subsequently loaded and run. Gels were incubated in the zymography development buffer containing 20 mM Tris (pH 7.4), 10 mM dithioerythreitol (DTE), and 2 mM calcium at room temperature for 24 h. Gels were stained with Coomassie brilliant blue. Upon destaining, calpain activity developed as clear bands against a dark background indicating proteolysis of casein.

### Immunoblot of Lp82

Water soluble proteins of rat lens (40 μg/well) were separated on a 12% SDS-gel. The proteins were electroblotted onto a nitrocellulose membrane (0.45 μm, Bio-Rad) under ice-cold temperature for one h at 100 V. The membrane was blocked overnight at 4 °C with 2% BSA (Bovine Serum Albumin, Fraction V prepared in Tris buffered saline-Tween-20). After rinsing the membrane with Tris buffered saline Tween (TBST), it was incubated for two h with 1:1000 diluted rabbit anti-Lp82 primary antibody. The membrane was washed three times (15 min each) with TBST and incubated with 1:5000 diluted goat anti-rabbit IgG secondary antibody coupled to alkaline phosphatase. The membrane was again washed three times (15 min each) with TBST buffer and developed with the BCIP/NBT substrate that developed into purple blue insoluble precipitates indicating the presence of Lp82. β-Actin was used as a loading control with rabbit anti-β-actin primary antibody at 1:1000 dilution.

### Isolation of water soluble protein fraction

Lenses were homogenized on ice in 1.0 ml of ice cold PBS (pH 7.4). Each homogenate was centrifuged at 10,000x g for 20 min at 4 °C and the precipitate was washed three times with the same buffer. The supernatant obtained was taken as the water soluble protein fraction.

### Sodium-dodecyl-sulfate polyacrylamide gel electrophoresis of water soluble protein

Sodium-dodecyl-sulfate polyacrylamide gel electrophoresis of water soluble protein (SDS-PAGE) was carried out in Hoeffer Slab gel apparatus using the method of Laemmli (1970) with some modifications [26]. A 12% gel of 7x8 cm and 1.0 mm thickness was used. The electrophoretic mobility depends on both molecular charge and size, so that the resulting protein pattern is characteristic of the specimen. All reagents and gels were made using Laemmli's system. The protein concentration of the samples was estimated and 20 μl (40 μg) was loaded in each well.

### Estimation of protein value

The protein content of the samples was determined by the method of Lowry et al [27] using bovine serum albumin as the standard.

### Statistical Analysis

All statistical calculations were carried out with the Statistical Package for Social Sciences (SPSS) software program (version 10.0 for Windows). The values are expressed as the mean±SD. The data were statistically analyzed using one-way analysis of variance (ANOVA) and significant difference of means was determined using Duncan's multiple range tests at the level of p<0.05 [28].

## Results

### Lens morphology

**Figure 1:**
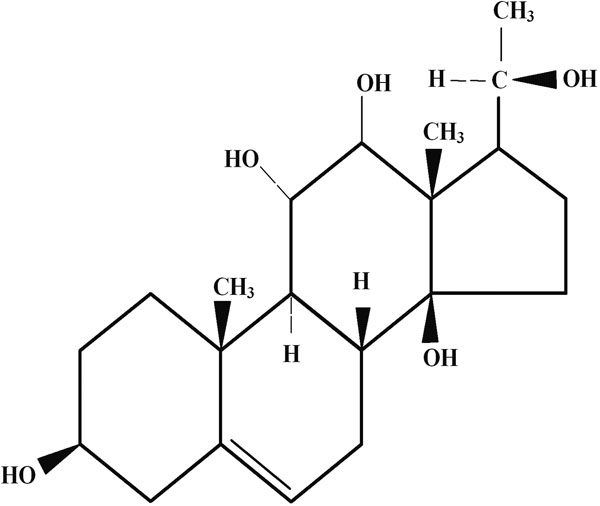
Structure of Drevogenin D. Drevogenin D is the bioactive component isolated from *Dregea volubilis*. Shown is the steroidal ring structure of Drevogenin D (3β, 11β, 12β, 14β, 20ζ- Pentahydroxy δ^5^ pregnene), a triterpenoid aglycone isolated from the leaves of *Dregea volubilis*.

**Figure 2:**
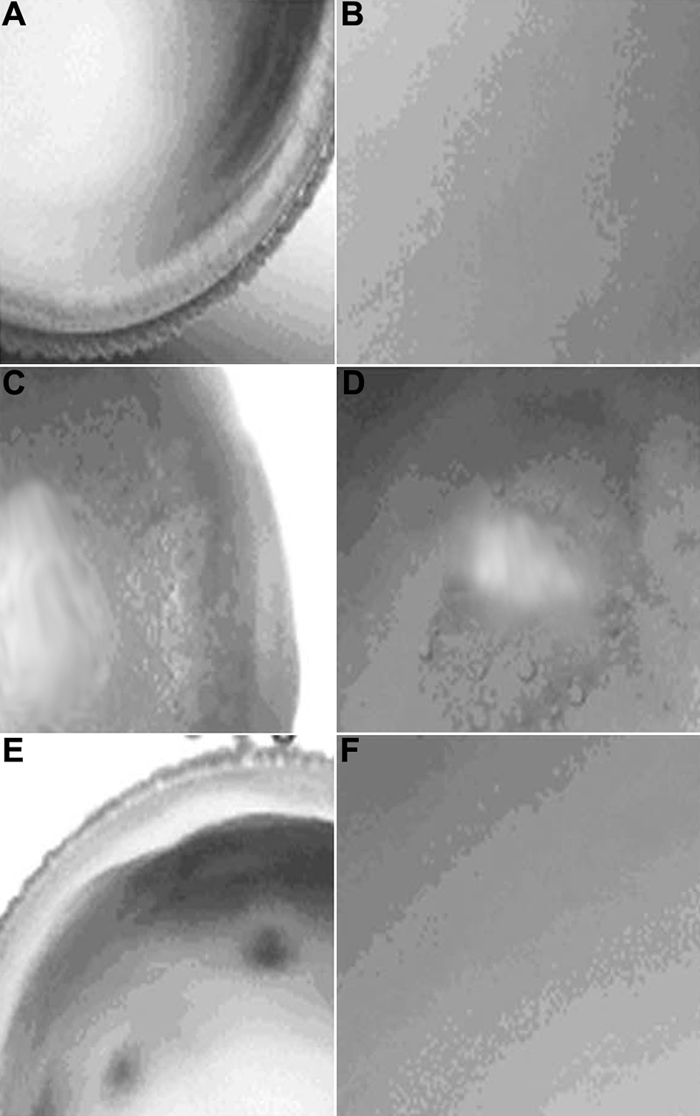
Rat lenses in various groups. **A** and **B** are normal lenses, **C** and **D** are selenite induced lenses, and **E** and **F** are selenite + Drevogenin D induced lenses. The magnifications of **A**, **C**, and **E** were 40X and the magnification of **B**, **D**, and **F** was 200X.

All the lenses in G-I were found to be transparent. Twenty-four h of incubation in the presence of sodium selenite produced a dense cortical vacuolization and opacification. The addition of Drevogenin D (Figure 1) to the culture medium prevented the formation of vacuoles and opacity to a greater extent. Six lenses (75%) of G-III were transparent when supplemented with Drevogenin D and the remaining lenses developed only lesser amount of cortical vacuolization and opacity (Figure 2).

### Activity of superoxide dismutase

**Figure 3:**
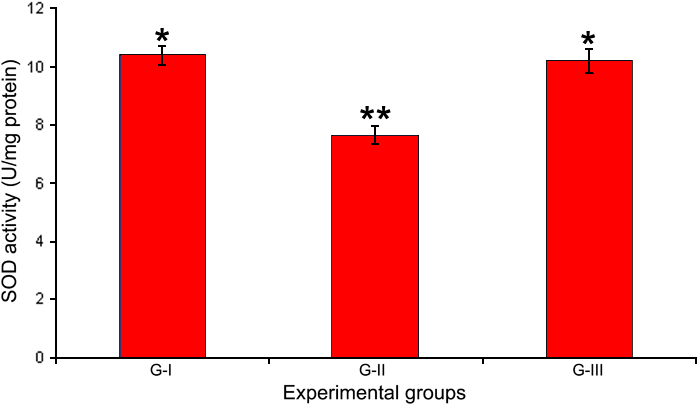
Activity of SOD in lens. Activity of the antioxidant enzyme SOD in the lens of experimental groups. Values are expressed as mean (n=8) ±SD. Different symbols indicate statistically significant difference between groups at p<0.05 using one-way ANOVA. Groupings are G-I: Control, G-II: Selenite-supplemented, G-III: Selenite-supplemented + Drevogenin D treated.

The activity of superoxide dismutase (SOD) was decreased following selenite administration, while treatment with Drevogenin D was found to maintain significantly higher levels of enzyme activity (Figure 3).

### Activity of Ca^2+^ ATPase and levels of Ca^2+^

**Figure 4:**
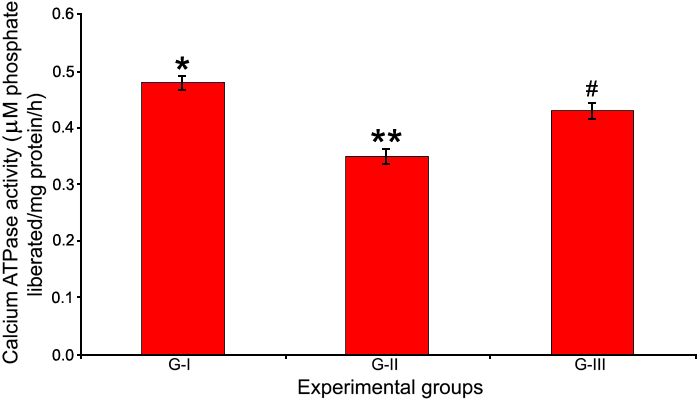
Activity of Ca^2+^ ATPase in lens. Activity of the ionic pump Ca^2+^ ATPase in the lens of experimental groups. Values are expressed as mean (n=8) ±SD. Different symbols indicate statistically significant difference between groups at p<0.05 using one-way ANOVA. Groupings are G-I: Control, G-II: Selenite-supplemented, G-III: Selenite-supplemented + Drevogenin D treated.

**Figure 5:**
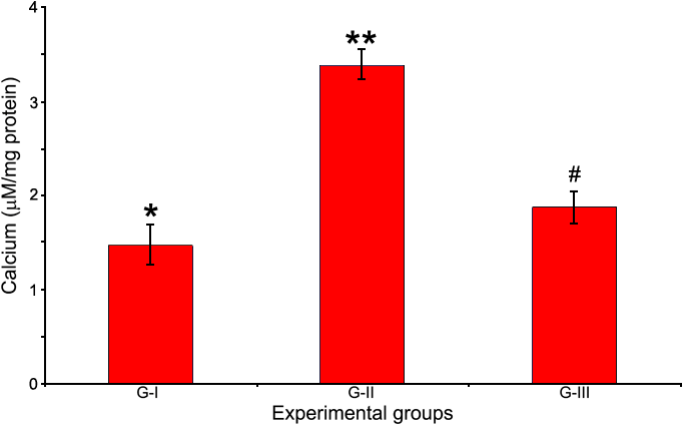
Levels of Ca^2+^ in lens. The ionic concentration of Ca^2+^ in the lens of experimental groups. Values are expressed as mean (n=8) ±SD. Different symbols indicate statistically significant difference between groups at p<0.05 using one-way ANOVA. Groupings are G-I: Control, G-II: Selenite-supplemented, G-III: Selenite-supplemented + Drevogenin D treated.

Activity of the membrane ionic pump, Ca^2+^ ATPase, was found to be decreased significantly following selenite induction whereas, treatment with Drevogenin D was found to maintain activity close to the normal levels (Figure 4). The levels of Ca^2+^ were significantly elevated in G-II following selenite induction compared to G-I and G-III. Drevogenin D treatment was observed to maintain the levels of Ca^2+^ in the lens close to the normal level (Figure 5).

### Levels of protein sulfhydryl content and thiobarbituric acid reacting substances

**Figure 6:**
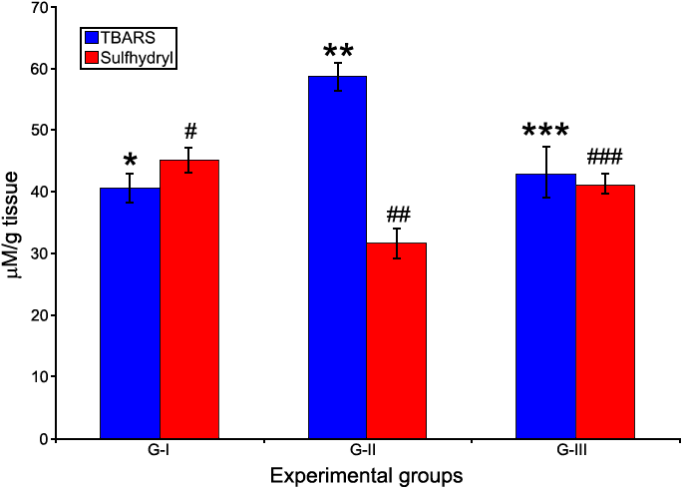
TBARS and sulfhydryl content in lens. The levels of TBARS (a lipid peroxidation product index) and Sulfhydryl content (an index of protein oxidation) in lens. Values are expressed as mean (n=8) ±SD. Different symbols indicate statistically significant difference between groups of each parameter at p<0.05 using one-way ANOVA. Groupings are G-I: Control, G-II: Selenite-supplemented, G-III: Selenite-supplemented + Drevogenin D treated.

The lens protein sulfhydryl content is an indicator of protein oxidation and was significantly reduced in G-II compared to G-I and G-III. Drevogenin D treatment maintained the levels of sulfhydryl content close to normal (Figure 6). TBARS, an indicator of lipid peroxidation, were significantly elevated following selenite induction in G-II whereas, Drevogenin D treatment showed a significant reduction in the levels of TBARS in G-III (Figure 6).

### Zymography and immunoblot

**Figure 7:**
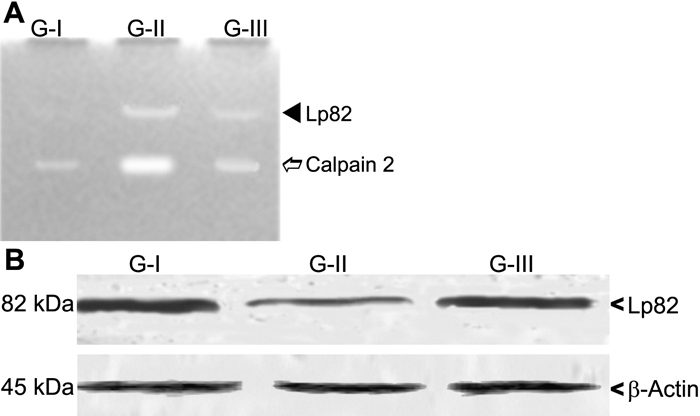
Casein zymography and immunoblot of calpains in lens. **A**: Casein zymographic analysis is shown where G-I deonotes normal lenses, G-II indicates selenite supplemented lenses, and G-III signifies selenite + Drevogenin D lenses. **B**: An immunoblot of Lp82 is illustrated where G-I signifies normal lenses, G-II denoted selenite-supplemented lenses, and G-III indicates selenite + Drevogenin D lenses. Actin was used as a normalizing control.

The zymogram for calpain exhibited activities of calpain 2 and Lp82 as caseinolytic clear bands. Lp82 gave an activity band close to the start of the gel while calpain 2 was further down. Drevogenin D treatment samples, G-III, showed a marked decline in the intensity of clearings but were marginally greater than G-I (Figure 7A). The presence of Lp82 proteins in the lens samples was visualized by an immunoblot (Figure 7B). G-II was found to show lower levels of Lp82 proteins as compared to G-I and G-III. Levels of the protein were comparable between G-I and G-III. Equal protein loading was confirmed by blotting the membrane with actin.

### Sodium-dodecyl-sulfate polyacrylamide gel electrophoresis of water soluble protein fraction

**Figure 8:**
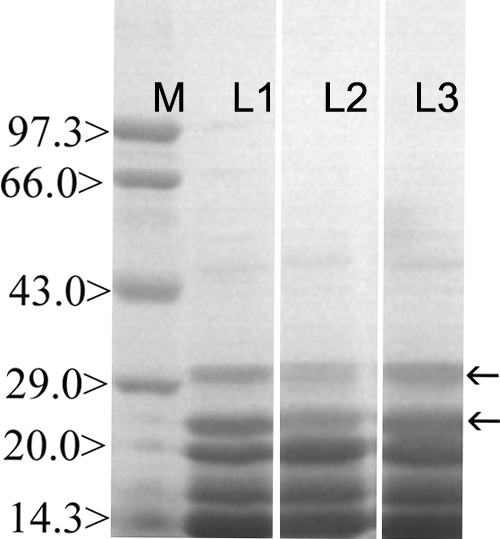
Sodium-dodecyl-sulfate polyacrimide gel electrophoresis of lens water soluble protein fraction. Lane M indicates the molecular weight marker, Lane 1 represents the protein profile of the normal lens while Lane 2 signifies the protein profile of the selenite-supplemented group, and Lane 3 denotes the protein profile of the Drevogenin D-treated group.

The SDS-PAGE profile of the water soluble lens proteins was carried out to visualize the effect of calpain-mediated proteolysis. G-I (Lane-I) showed the normal protein profile of the WSF. Two bands in G-II (Lane-2) were found to have lower expression intensity in WSF and correspond to 22.0 kDa and 30.5 kDa, respectively. These bands in G-III (Lane-2) showed expression near to normal levels found in G-I (Figure 8).

## Discussion

Oxidative stress, which occurs due to the formation of free radicals in the cell as a consequence of both enzymatic and non-enzymatic reactions, has been established as a major factor involved in the development of most types of cataract [29]. Selenite-induced cataract has received much attention and was worked upon as a model system for oxidative stress-induced cataract. The mechanism of selenite cataract involves oxidation of sulfhydryl groups of proteins and calcium dependent activation of proteases [30]. Oxidative stress-induced lipid peroxidative damage of membranes might be a contributing factor in the loss of Ca^2+^ ATPase activity and accumulation of Ca^2+^, which in turn leads to the activation of lens calpains and subsequent proteolytic degradation of lens soluble proteins in selenite cataract.

Previous reports from India have shown that phytochemicals play an important role in the prevention of oxidative stress-induced cataract [31,32]. Our earlier studies have indicated Drevogenin D, a triterpenoid aglycone (Figure 1), to possess excellent antioxidant potential and alleviate selenite-induced cataractogenesis in animal models [under publication]. In the present study, organ-cultured rat lens was used as the model to determine the protective effects of Drevogenin D against selenite-induced cataractogenesis. Cortical vacuolization and opacification are characteristic of in vitro selenite cataract while it is a nuclear cataract in selenite-induced in vivo rat pup model. Although the opacifications developed are different, the underlying mechanisms are found to be similar [9,33].

Calcium is essential for various lens fiber cell processes including its differentiation [34]. The levels of the divalent cation Ca^2+^ in the lens is maintained at submicromolar range and is lower than that of the aqueous humor [7]. It has also been found that alterations in the homeostasis of lenticular Ca^2+^ have been implicated in cataractogenesis [35]. In this study, activity of Ca^2+^ ATPase was significantly decreased by selenite administration with a corresponding increase in Ca^2+^. Treatment with Drevogenin D was observed to maintain the activity of Ca^2+^ ATPase and levels of Ca^2+^ close to normal range (Figure 4 and Figure 5). The level of Ca^2+^ is maintained by Ca^2+^ ATPase, which counteracts the inward passive diffusion of Ca^2+^ [36]. Ca^2+^ ATPase is a major factor involved in maintaining lenticular Ca^2+^ levels and loss of its activity could explain the rise in Ca^2+^. Earlier reports have shown that Ca^2+^ ATPases are particularly sensitive to oxidation and that oxidative damage leads to decreased activity of these enzymes, resulting in elevated levels of Ca^2+^ in the lens [37]. Ca^2+^ ATPases are also reported to be sensitive to membrane lipid order and their decreased activity may be related to structural changes in membrane lipids [38]. Studies also shown that lens lipids have a high affinity to bind Ca^2+^ and any alteration may decrease its capacity, which results in elevated levels of Ca^2+^ [38,39]. These findings are in agreement with our observation that selenite-induced oxidative stress resulted in higher levels of lipid peroxidation, loss of activity of Ca^2+^ ATPase, and accumulation of Ca^2+^ in the lens. The lower levels of Ca^2+^, higher activity of Ca^2+^ ATPase, and decreased levels of lipid peroxidation in the lens of the Drevogenin D-treated group could be attributed to its antioxidant protection against selenite-induced oxidative stress.

Calpains are Ca^2+^-activated neutral proteases found in all tissues. In spite of their important regulatory functions under normal physiological conditions, accumulated levels of Ca^2+^ are observed to result in the proteolysis of crystallins associated with cataractogenesis. Though not found in humans, Lp82 are of significant importance in rodent cataractous models [40]. In the present study, zymographic results of Lp82 and the ubiquitous calpain 2 showed bands of greater intensity in the selenite-induced group. Activation of calpains was found to be in tandem with the rise in levels of Ca^2+^ as described above (Figure 7A). Treatment with Drevogenin D was found to exhibit a lower activation index, which was comparable with the level of Ca^2+^ observed. Hence, it could be inferred that the near normal levels of Ca^2+^ and Ca^2+^ ATPase activity following Drevogenin D treatment has a direct bearing on the observed decrease in the activation of Lp82 and calpain 2. The immunoblot of Lp82 was carried out to assess the level of its protein in relation to calpain activation. Major loss of Lp82 protein has been reported during selenite-induced cataract, which might be explained by the process of auto-proteolysis [41]. The level of Lp82 protein was found to be lowered in the selenite-induced group that exhibited a higher level of calpain activity while Drevogenin D-treated group exhibited protein levels almost similar to the normal exhibited in G-I (Figure 7B).

Crystallins, the major structural proteins of the lens, are excellent substrates for lenticular calpains [42]. The elevated levels of activity for lenticular calpains contribute to cataractogenesis [43]. The ability of calpain 2 as well as Lp82 to proteolyse α- and β-crystallins has been demonstrated in mice [44]. Earlier studies have reported that β-crystallin is more susceptible with calpain 2 and Lp82 sharing similar proteolysis pattern whereas α-crystallin had different cleavage sites for the two calpains [45]. Calpain-induced truncation of crystallins is a major cause of altered protein-protein interaction, which leads to protein precipitation. In our study, the SDS-PAGE pattern of soluble proteins showed a decrease in the intensity of two bands corresponding to 22.0 and 30.5 kDa in the selenite-induced group (Figure 8). These results suggested that proteolytic insolubilization of these bands may be due to excessive calpain activity in this group (Figure 7A). The Drevogenin D-treated group had the expression of these bands at normal levels while the activation of calpains was only marginal. Hence, it might be inferred that these bands correspond to the proteolysed crystallins in the selenite-induced group. Lenticular fodrin are also degradative substrates for calpain and their degradation has been associated with cataractogenesis [46]. Fodrin/spectrin proteolysis could also be a possible event in this study in the development of lens opacification but the SDS-PAGE profile showed no significant change at the molecular range, which was expected for fodrin while the change was significant at a lower molecular range suggesting crystallin degradation as the major event in calpain degradation.

Oxidative stress induced by selenite and its mitigation by Drevogenin D treatment was assessed by measuring the activity of SOD (Figure 3), levels of protein sulfhydryl content, and TBARS (Figure 6). The activity of SOD and protein sulfhydryl content was significantly lowered in the selenite-induced group while the levels of TBARS showed a significant increase. The loss of SOD activity could be expected due to the oxidation of sulfhydryl groups [31] and loss of membrane integrity. The Drevogenin D-treated group was found to exhibit the activities of SOD, sulfhydryl content, and TBARS near the normal level. Similar results were reported by us as well as other groups [16,33,47]. Mitochondria are rich source of Ca^2+^ and also the source of ATP in cells. Oxidative damage of mitochondria has been shown to increase cellular Ca^2+^ levels [48,49] and contribute to lens fiber cell globulization in vitro [50]. The protective effect of Drevogenin D could also be attributed to its mitochondrial protective activity by virtue of its ability to prevent oxidative stress and preoxidative damage of mitochondrial membrane, thereby keeping the Ca^2+^ level normal and preventing calpain activation.

These results suggest that Drevogenin D treatment is effective in protecting the lens proteins by maintaining Ca^2+^ ATPase activity, preventing an accumulation of calcium thus calpain activation, and prevention of protein oxidation and lipid peroxidation, which are all results of selenite induction, are supportive of the anticataractogenic potential of the compound. In our study Drevogenin D acts as an antioxidant to prevent critical sulfhydryl oxidation of Ca^2+^ ATPase and thereby, controlling the levels of calcium. Our results are found to be in agreement with the reported data [33]. The antioxidant property of Drevogenin D was already established in our laboratory (data under publication). This is the first study reported on the effect of a triterpenoid in general and of Drevogenin D in particular to protect against selenite-induced cataractogenesis.

## References

[1] Javitt JC Wang F West SK Blindness due to cataract: epidemiology and prevention. Annu Rev Public Health 1996 17 159 77 8724222 10.1146/annurev.pu.17.050196.001111

[2] Nirmalan PK Krishnadas R Ramakrishnan R Thulasiraj RD Katz J Tielsch JM Robin AL Lens opacities in a rural population of southern India: the Aravind Comprehensive Eye Study. Invest Ophthalmol Vis Sci 2003 44 4639 43 14578379 10.1167/iovs.03-0011

[3] Ughade SN Zodpey SP Khanolkar VA Risk factors for cataract: a case control study. Indian J Ophthalmol 1998 46 221 7 10218305

[4] Hightower KR David LL Shearer TR Regional distribution of free calcium in selenite cataract: relation to calpain II. Invest Ophthalmol Vis Sci 1987 28 1702 6 2820891

[5] Duncan G Williams MR Raich RA Calcium, cell signaling and cataract. Prog Retin Eye Res 1994 13 623 52

[6] Duncan G Webb SF Dawson AP Bootman MD Elliott AJ Calcium regulation in tissue-cultured human and bovine lens epithelial cells. Invest Ophthalmol Vis Sci 1993 34 2835 42 8360017

[7] Galvan A Louis CF Calcium regulation by lens plasma membrane vesicles. Arch Biochem Biophys 1988 264 472 81 2840857 10.1016/0003-9861(88)90312-8

[8] Churchill GC Louis CF Imaging of intracellular calcium stores in single permeabilized lens cells. Am J Physiol 1999 276 C426 34 9950770 10.1152/ajpcell.1999.276.2.C426

[9] Shearer TR Ma H Fukiage C Azuma M Selenite nuclear cataract: review of the model. Mol Vis 1997 3 8 http://www.molvis.org/molvis/v3/a8/9238097

[10] Hamakubo T Kannagi R Murachi T Matus A Distribution of calpains I and II in rat brain. J Neurosci 1986 6 3103 11 3021924 10.1523/JNEUROSCI.06-11-03103.1986 PMC6568489

[11] Shih M Ma H Nakajima E David LL Azuma M Shearer TR Biochemical properties of lens-specific calpain Lp85. Exp Eye Res 2006 82 146 52 16054132 10.1016/j.exer.2005.06.011

[12] Ma H Hata I Shih M Fukiage C Nakamura Y Azuma M Shearer TR Lp82 is the dominant form of calpain in young mouse lens. Exp Eye Res 1999 68 447 56 10192802 10.1006/exer.1998.0625

[13] David LL Azuma M Shearer TR Cataract and the acceleration of calpain-induced beta-crystallin insolubilization occurring during normal maturation of rat lens. Invest Ophthalmol Vis Sci 1994 35 785 93 8125740

[14] Stitt AW Advanced glycation: an important pathological event in diabetic and age related ocular disease. Br J Ophthalmol 2001 85 746 53 11371498 10.1136/bjo.85.6.746 PMC1723990

[15] Jacques PF Chylack LT Jr Epidemiologic evidence of a role for the antioxidant vitamins and carotenoids in cataract prevention. Am J Clin Nutr 1991 53 352S 5S 1985409 10.1093/ajcn/53.1.352S

[16] Grassmann J Terpenoids as plant antioxidants. Vitam Horm 2005 72 505 35 16492481 10.1016/S0083-6729(05)72015-X

[17] Chopra RN, Nayar SL, Chopra IC. Glossary of Indian Medicinal Plants. New Delhi: CSIR; 1999.

[18] Lija Y Biju PG Reeni A Cibin TR Sahasranamam V Abraham A Modulation of selenite cataract by the flavonoid fraction of Emilia sonchifolia in experimental animal models. Phytother Res 2006 20 1091 5 17009203 10.1002/ptr.2005

[19] Sahu NP Panda N Mandal NB Banerjee S Koike K Nikaido T Polyoxypregnane glycosides from the flowers of Dregea volubilis. Phytochemistry 2002 61 383 8 http://www.ncbi.nlm.nih.gov/sites/entrez?cmd=Retrieve&db=PubMed&list_uids=12377230&dopt=Abstract 12377230 10.1016/s0031-9422(02)00260-1

[20] Yoshimura S Narita H Hayashi K Mitsuhashi H Studies on the constituents of Asclepiadaceae plants. LIX. The structures of five new glycosides from Dregea volubilis (L.)Benth. Chem Pharm Bull (Tokyo) 1985 33 2287 93 10.1248/cpb.31.3971 6671238

[21] Kakkar P Das B Viswanathan PN A modified spectrophotometric assay of superoxide dismutase. Indian J Biochem Biophys 1984 21 130 2 6490072

[22] Rorive G Kleinzeller A Ca2+-activated ATPase from renal tubular cells. Methods Enzymol 1974 32 303 6 4280488 10.1016/0076-6879(74)32031-9

[23] Altomare E Grattagliano I Vendemaile G Micelli-Ferrari T Signorile A Cardia L Oxidative protein damage in human diabetic eye: evidence of a retinal participation. Eur J Clin Invest 1997 27 141 7 9061308 10.1046/j.1365-2362.1997.780629.x

[24] Niehaus WG Jr Samuelsson B Formation of malonaldehyde from phospholipid arachidonate during microsomal lipid peroxidation. Eur J Biochem 1968 6 126 30 4387188 10.1111/j.1432-1033.1968.tb00428.x

[25] Raser KJ Posner A Wang KK Casein zymography: a method to study mu-calpain, m-calpain, and their inhibitory agents. Arch Biochem Biophys 1995 319 211 6 7771786 10.1006/abbi.1995.1284

[26] Laemmli UK Cleavage of structural proteins during the assembly of the head of bacteriophage T4. Nature 1970 227 680 5 5432063 10.1038/227680a0

[27] Lowry OH Rosebrough NJ Farr AL Randall RJ Protein measurement with the Folin phenol reagent. J Biol Chem 1951 193 265 75 14907713

[28] Steel RG, Torrie JH, Dickey DA. Principles and Procedures of Statistics:A Biometrical Approach. New York: McGraw Hill; 1996.

[29] Spector A Oxidative stress-induced cataract: mechanism of action. FASEB J 1995 9 1173 82 7672510

[30] Boscia F Grattagliano I Vendemiale G Micelli-Ferrari T Altomare E Protein oxidation and lens opacity in humans. Invest Ophthalmol Vis Sci 2000 41 2461 5 10937554

[31] Thiagarajan G Chandani S Harinarayana Rao S Samuni AM Chandrasekaran K Balasubramanian D Molecular and cellular assessment of ginkgo biloba extract as a possible ophthalmic drug. Exp Eye Res 2002 75 421 30 12387790

[32] Thiagarajan G Venu T Balasubramanian D Approaches to relieve the burden of cataract blindness through natural antioxidants: use of Ashwagandha (Withania somnifera) Curr Sci 2003 85 1065 71

[33] Gupta SK Srivastava S Trivedi D Joshi S Halder N Ocimum sanctum modulates selenite-induced cataractogenic changes and prevents rat lens opacification. Curr Eye Res 2005 30 583 91 16020293 10.1080/02713680590968132

[34] Wride MA Cellular and molecular features of lens differentiation: a review of recent advances. Differentiation 1996 61 77 93 8983174 10.1046/j.1432-0436.1996.6120077.x

[35] Gupta PD Johar K Vasavada A Causative and preventive action of calcium in cataracto-genesis. Acta Pharmacol Sin 2004 25 1250 6 15456524

[36] Liu L Paterson CA Borchman D Regulation of sarco/endoplasmic Ca2+ -ATPase expression by calcium in human lens cells. Exp Eye Res 2002 75 583 90 12457870 10.1006/exer.2002.2049

[37] Ahuja RP Borchman D Dean WL Paterson CA Zeng J Zhang Z Ferguson-Yankey S Yappert MC Effect of oxidation on Ca2+ -ATPase activity and membrane lipids in lens epithelial microsomes. Free Radic Biol Med 1999 27 177 85 10443934 10.1016/s0891-5849(99)00068-4

[38] Borchman D Lamba OP Yappert MC Structural characterization of lipid membranes from clear and cataractous human lenses. Exp Eye Res 1993 57 199 208 8405186 10.1006/exer.1993.1115

[39] Tang D Borchman D Schwarz AK Yappert MC Vrensen GF van Marle J DuPre DB Light scattering of human lens vesicles in vitro. Exp Eye Res 2003 76 605 12 12697424 10.1016/s0014-4835(03)00026-5

[40] Nakamura Y Fukiage C Shih M Ma H David LL Azuma M Shearer TR Contribution of calpain Lp82-induced proteolysis to experimental cataractogenesis in mice. Invest Ophthalmol Vis Sci 2000 41 1460 6 10798663

[41] Shearer TR Ma H Shih M Hata I Fukiage C Nakamura Y Azuma M Lp82 calpain during rat lens maturation and cataract formation. Curr Eye Res 1998 17 1037 43 9846621 10.1076/ceyr.17.11.1037.5232

[42] Shearer TR Shih M Mizuno T David LL Crystallins from rat lens are especially susceptible to calpain-induced light scattering compared to other species. Curr Eye Res 1996 15 860 8 8921229 10.3109/02713689609017627

[43] Huang Y Wang KK The calpain family and human disease. Trends Mol Med 2001 7 355 62 11516996 10.1016/s1471-4914(01)02049-4

[44] Biswas S Harris F Singh J Phoenix D Role of calpains in diabetes mellitus-induced cataractogenesis: a mini review. Mol Cell Biochem 2004 261 151 9 15362498 10.1023/b:mcbi.0000028750.78760.6f

[45] David LL Shearer TR Shih M Sequence analysis of lens beta-crystallins suggests involvement of calpain in cataract formation. J Biol Chem 1993 268 1937 40 8420967

[46] Kilic F Trevithick JR Modelling cortical cataractogenesis. XXIX. Calpain proteolysis of lens fodrin in cataract. Biochem Mol Biol Int 1998 45 963 78 9739461 10.1002/iub.7510450514

[47] Doganay S Borazan M Iraz M Cigremis Y The effect of resveratrol in experimental cataract model formed by sodium selenite. Curr Eye Res 2006 31 147 53 16500765 10.1080/02713680500514685

[48] Vlessis AA Mela-Riker L Selenite-induced NAD(P)H oxidation and calcium release in isolated mitochondria: relationship to in vivo toxicity. Mol Pharmacol 1987 31 643 6 3600609

[49] Crawford DR Wang Y Schools GP Kochheiser J Davies KJ Down-regulation of mammalian mitochondrial RNAs during oxidative stress. Free Radic Biol Med 1997 22 551 9 8981048 10.1016/s0891-5849(96)00380-2

[50] Wang L Christensen BN Bhatnagar A Srivastava SK Role of calcium-dependent protease(s) in globulization of isolated rat lens cortical fiber cells. Invest Ophthalmol Vis Sci 2001 42 194 9 11133867

